# Long-Term Effect of Gluten-Free Diets on Nutritional Status, Body Composition, and Associated Factors in Adult Saudi Females with Celiac Disease

**DOI:** 10.3390/nu14102090

**Published:** 2022-05-17

**Authors:** Aeshah Ibrahim Alhosain, Ghedeir M. Alshammari, Barakat Lafi Almoteri, Mohammed A. Mohammed, Manal Abdulaziz Binobead, Mohammed Abdo Yahya

**Affiliations:** 1 Department of Food Science and Nutrition, College of Food and Agricultural Sciences, King Saud University, Riyadh 11451, Saudi Arabia; 439204473@student.ksu.edu.sa (A.I.A.); muhammed-awad@hotmail.com (M.A.M.); mbinobead@ksu.edu.sa (M.A.B.); mabdo@ksu.edu.sa (M.A.Y.); 2 Department of Gastroenterology, Buraidah Central Hospital, Ministry of Health, Buraydah 52361, Saudi Arabia; vv4x@hotmail.com

**Keywords:** celiac, dietary intake, body composition, body mass index, body fat

## Abstract

This cross-sectional study examines the influence of long-term gluten-free diet (GFD) consumption on nutritional status, body composition, and associated factors in adult Saudi females with celiac diseases (CD). Fifty-one patients who have been diagnosed with CD and have been on GFD for more than 1 year were included in this study where data regarding their dietary pattern, as well as a complete analysis of their anthropometric parameters, vitamins B12 and D levels, and complete blood count (CBC), were collected. Data have shown that all included patients showed a reduced intake in all micro and macro-nutrients, as well as vitamin D, folate, calcium, and iron. However, the vast majority of all measured hematological parameters and blood indices were within the expected reference range. In addition, 51%, 43.1%, and 60.8% of the patients showed low waist/hip ratio (WHR), decreased level of total body fat (BF), and decreased level of visceral fat (VF), respectively, whereas 33.3% were slim. The poor educational level and some psychosocial factors were associated with the poor nutritional status of the patients. In conclusion, the GFD-dependent intake by female patients with CD adversely affects their nutritional intake and anthropometric indices and leads to a deficiency in major nutrients, vitamins, and ions.

## 1. Introduction

Celiac disease (CD) is a chronic inflammatory autoimmune disorder that affects the gut in response to gluten intolerance and increased gluten intake in predisposed individuals [1]. Currently, the prevalence of CD is rapidly increasing worldwide, as well as in the Arabian region, where the Kingdom of Saudi Arabia (KSA) has the highest rate (3.2%) [2]. In addition, clinical reports also show that the prevalence of CD is higher in females as compared to males [2]. Yet, the gluten-free diet (GFD) intake remains the most available therapy to alleviate intestinal damage and reduce nutrient absorption in those patients [3]. 

Unfortunately, nutritional and clinical studies in CD patients have shown that the complete dependence on and long-term intake of GFD, as a sole source of diet is associated with a reduced energy intake and several adverse health effects, such as deficiencies in essential nutrients, vitamins, and ions, as well as an increased risk of obesity, diabetes mellitus (DM), metabolic disorder (Mets), and cardiovascular disorders (CVDs) [4,5,6,7,8,9]. Indeed, several authors have shown that GFD contains an insufficient amount of carbohydrates, fiber, some minerals, and vitamins due to refining fiber grains and mixing commercial GFD with high fats, saturated and hydrogenated fatty acids [8,9]. Additionally, patients having CD are at increased risk of developing low bone mineral density (BMD) and bone alterations due to the reduced intake of vitamin D and calcium (Ca^2+^), as they are at a high risk of developing lactose intolerance [3,10,11]. This enforced the importance of replacement therapy and the use of lactose-free products [3,10]. In addition, CD patients also have impaired normal bone metabolism due to the lack of fat-soluble vitamins [11]. According to Valdimarsson et al. [12], the low BMD in CD patients is associated secondary hyperparathyroidism and reduced levels of 25-hydroxy-vitamin D, which remained significantly low after 3 years following the GFD. 

Furthermore, regularly physically active people have a higher bone mass and fewer fractures than those who are sedentary [13]. It has been reported that male and female patients with CD who follow a GFD and in complete remission of the disease have a low body mass index (BMI) and body fat content compared to normal individuals [14,15]. Additionally, some aspects of the GFD profile may be associated with an increased risk of other forms of chronic disorders. Within this view, Tovoli et al. [16] demonstrated that more than one-third of CD patients who followed a GFD had a three-fold increased risk of developing nonalcoholic fatty liver disease (NAFLD). Furthermore, they have shown that dietary advice tailored to the patient should help CD patients with NAFLD to achieve an appropriate nutritional intake, while lowering the risk of long-term liver problems. However, despite GFD being associated with important nutrient deficiency, it may have protective effects on other systems. Indeed, the adherence to GFD reduced anti-ganglioside antibodies and neurological disorders in CD patients [17]. However, restriction on GFD did not alter the prevalence of atopic rash in Italian CD patients [18].

In KSA, there is still a lack of knowledge on the adverse health of the adherence to GDF in CD patients. Therefore, the current research was conducted to study the effects of GFD on the nutritional status, body composition, and associated factors of Saudi adult females with celiac disease.

## 2. Materials and Methods

### 2.1. Subjects

This was a cross-sectional study that was conducted at Buraidah Central Hospital, KSA, between August and December 2021 on adult Saudi female volunteers who had been previously diagnosed with CD. The total number of participants was 51. Inclusion criteria were a female patient with an average age of 33, tested positive for the anti-tissue transglutaminase IgA class antibodies (anti-tTG antibodies), diagnosed with CD, on GFD for at least one year, no intake of any nutritional supplements, and had no other comorbidities. Other exclusion criteria were females who were pregnant or breastfeeding, as well as those with immunoglobulin A (IgA) deficiency.

### 2.2. Analysis of Dietary Intake 

Adult Saudi females diagnosed with CD and relying on GFD for more than a year were interviewed face to face, and food intake was tracked using structural questionnaires approved by an expert committee for the previous three days over 24 h. The questionnaires focused on the number and kinds of meal intake per day and their frequency of intake. The questionnaire was conducted with the help of a family to facilitate the description of food intake for patients. A dietitian trained all participants for 7 days to record all food consumed. Participants were asked to weigh all food and drink they consumed and to provide a detailed description of each food, including methods of preparation and recipes, when possible. The dietitian reviewed the three days’ intake with participants at the second visit to check for errors or omissions and to estimate the amount of food eaten outside the home using standard household measurements. Thereafter, total food and beverage consumption was calculated and recorded.

The nutritional data collected were analyzed by the ESHA computer program to convert food intake into energy and nutrients. The recommended dietary intake (DRI) of energy and nutrients reported by the World Health Organization (WHO) and Food and Agriculture Organization (FAO) dietary guidelines for a different population was used as a standard to compare the results obtained.

### 2.3. Anthropometric Measurement

Anthropometric parameters were measured by a body composition analyzer (model ACCUNIQ BC360; ELVAS Healthcare Inc., Daejeon, Korea). Measurements were taken while patients were standing, with electrodes placed on both hands and feet. The device was connected to an ultrasonic height meter, and body weight, waist circumference (WC), BMI, body fat (BF), visceral fat (VF), and waist-to-hip ratio (WHR) were automatically calculated. Patients’ palms and soles were cleaned after each measurement. In addition, patients were not allowed to eat or have any drink for 4 h before testing. The participants were afforded adequate privacy. BMI (kg/m^2^) was performed to assess the status of body weight and classified as described by WHO [19] as the following: (1) <18.5: underweight, (2) 18.5–24.9: normal weight, (3) 25–29.9: overweight, and (4) ≥30: obese. Moreover, three grade levels of obesity were considered: (1) BMI = 30–34.9: Grade 1, (2) BMI = 35–39.9: Grade 2, and (3) BMI ≥ 40: Grade 3 (extreme obesity). As described by Kyle et al. [20], the percentages of the BF (BF%) of each of the patients were classified as (1) <11: low, (2) 11–21.9%: normal, (3) 22–27: high, (4) >27: very high. The classification of the VF content was as the following: (1) <10 level: low, (2) 10–14.9 level: normal, (3) 15–20 level: high, and (4) > 20 level: very high. Waist-hip ratio (WHR) was classified according to study of Nishida et al. [21] as: (1) low (≤0.80), (2) normal (= 0.81–0.85), and (3) high (≥0.86). The classification based on the WHR followed the study of classification by Ashwell et al. [22] as: (1) extremely slim (≤0.34), (2) slim: (0.35–0.41), (3) normal (0.42–0.48), (4) overweight: (0.49–0.53), (5) very overweight (0.54–0.57), and (6) obese (≥0.58)

### 2.4. Blood Parameters

A fasting venous blood sample was collected from each patient in EDTA containing tubes. Patients’ complete blood count (CBC) was carried out in the laboratories of Buraidah Central Hospital using a Sysmex blood counter (model XN1000-Cobas E411, Sysmex-Rauch-Hitachi, Tokyo, Japan). The plasma levels of vitamin B12 (VB12) and vitamin D (VD) were measured using the dimension EXL™ 200 integrated chemistry system (Siemens Healthcare, Erlangen, Germany). All measurements were performed as per each machine manufacturer’s instructions. The measured parameters and their reference values for adults are shown in Table 1. 

### 2.5. Ethical Approval

The research protocol was approved by the Ethics Committee for Human Studies in Qassim No. (1844070-1442), and the work was carried out under the legal requirements and guidelines for good clinical practice. Written consent to participate in the study was collected from all volunteers. Anonymity and protection of personal data were ensured for all participants.

### 2.6. Data Analysis

The data were analyzed using the statistical package for social sciences (SPSS Inc., Chicago, IL, USA; version 20). The mean values of the females’ nutrient intake and DRI were correlated using a Student’s t-test. The significant level was set at ** *p* ≤ 0.01; * *p* ≤ 0.05. The relationship between demographic characteristics, nutritional proxies (WHR, WHtR, BMI, BF, and VF), as well as nutritional indices (BMI, BF, and VF), and blood measurements were investigated using Spearman correlation coefficients. A simple regression analysis was used to determine the effect of independent variables on the levels of dependent variables. 

## 3. Results

### 3.1. Nutrient Intake and Blood Parameters 

Table 2 shows the nutrient intake compared to DRI values for Saudi adult females with CD. According to the data, the actual intake of females with CD did not cover the DRI of all nutrients, and the level of nutrient intake was significantly (*p* ≤ 0.01) lower than that of the DRI. The daily intake of iron, phosphorus, copper, calcium, folate, and vitamin A was significantly lower than their DRI (Table 2). Table 3 shows the results of the CBC in all participating patients. In accordance, more than 90% of the female CD patients had normal WBC, RBC, RDW-SD, MPV, P-LCR, and vitamin B12, but showed a significant reduction in PDW values. In addition, 54.9% of the patients had significantly low levels of VD (Table 3) and all participants had a high level of PCT (Table 3). For the remaining blood parameters, 50–80% of participants showed normal values.

### 3.2. Anthropometric Measurements

The BMI, WHR, BF, VF, and waist-to-height ratio (WHtR) of the females were calculated, and the data are presented in Table 4. The results showed that 9.8% of the females were underweight and had reduced BMI. However, 51.0% of the patients had a normal BMI, while 27.5% of them were overweight. Additionally, 3.9% and 7.8% of the participants were obese and were classified into obesity levels I and II, respectively. The results indicated that the females with normal BMI exceeded the other groups. 

Since adipose tissue distribution differed between individuals, BMI as an index of a respondent’s obesity does not always correctly show the degree of a respondent’s visceral fat level. Therefore, in this study, in addition to BMI as an indicator, BF, WHR, VF, and WHtR were used to investigate the nutritional status of the females. As shown in Table 4 and according to the body composition analyzer, 51.0% of the females had a low WHR, only 5.9% had a normal WHR, and 43.1% had high levels. The majority of the females have a decreased BF level (43.1%), with a very low percentage with very high BF (13.7%). Additionally, the majority of females had decreased values of VF (60.8%) compared to those with high VF (13.7%). About 5.9% of the females were extremely slim, 33.3% were slim, and 29.4% were extremely overweight.

### 3.3. Factors Associated with Nutritional Status

Some of the associated factors that are expected to influence the nutritional status of Saudi adult females with CD are shown in Table 5. The data analysis between the females’ anthropometric proxies as dependent variables and demographic characteristics using both Spearman correlation coefficient and simple regression analysis showed either positive or negative correlations between the variables. WHR, WHtR, BMI, BF, and VF were used as proxies for the females’ nutritional status. The females’ age was significantly and favorably correlated with WHR as well as WHtR, BMI, BF, and VF (*p* ≤ 0.01), with a high effect observed on WHR (β** = 2.87, r^2^ = 0.27) compared to other indicators as indicated by simple regression analysis. The type of residence of the females was significant (*p* ≤ 0.01) and favorably affected all nutritional indicators with a greater effect on WHtR (β** = 1.028, r^2^ = 0.182). On the other hand, the occupation of the females significantly (*p* ≤ 0.01) and favorably correlated with all dependent variables with a greater association with BMI (β** = 2.081, r^2^ = 0.825). The marital status of the females with CD was favorably and significantly (*p* ≤ 0.01; 0.05) associated with WHR, BMI, BF, and VF (*p* ≤ 0.05), with a greater association with VF (β** = 0.056, r^2^= 0.179) than other proxies. However, the education level was found to harm all proxies with a high effect on WHtR (β* = 0.284, r^2^ = 0.205). 

**Figure 1:**
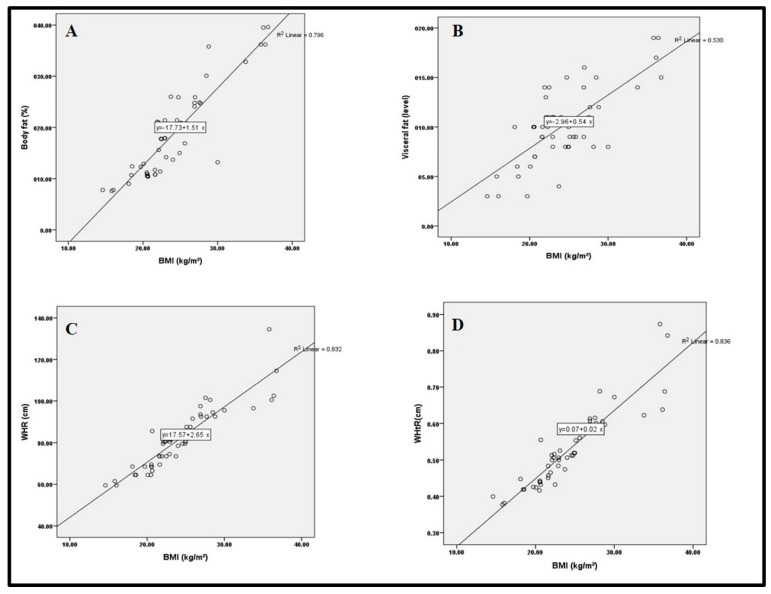
Regression models of (**A**) correlation between body mass index (BMI) and percentage body fat in females with CD y = 17.73 + 1.51x, r^2^ = 0.796; (**B**) correlation between body mass index (BMI) and level of visceral fat in women with CD y = 2.96 + 0.54x, r^2^ = 0.530; (**C**) correlation between body mass index (BMI) and (WHR) in women with CD y = 17.57 + 2.65x, r^2^ = 0.832; (**D**) correlation between body mass index (BMI) and (WHTR) in women with CD y = 0.07+ 0.02x, r^2^ = 0.836.

Further to the study factors associated with the females’ nutritional status, a correlation was carried out between the females’ nutritional indices (BMI, BF, and VF) as dependent variables and some psychosocial factors as independent variables. As shown in Table 6, enquires were either adversely or favorably associated with the females’ nutritional indices. The study observed that the females concerned about the emergence of a health issue related to CD and that they might accidentally eat or drink products containing gluten did not affect their nutritional indices. However, depression and anxiety, the avoidance of social activities, and the cost of GFD or inability to work were adversely and significantly (*p* ≤ 0.05) correlated with both BF and VF. As shown in Figure 1, a strong correlation between BMI and BF (r^2^ = 0.794), VF (r^2^ = 0.538), WHR (r^2^ = 0.832) and WHtR (r^2^ = 0.836) was observed.

## 4. Discussion

The present study investigated the long-term effect of GFD on nutritional status, body composition, and associated factors in adult Saudi females with CD. The nutrient intake of females with CD compared to the dietary recommended intake (DRI), deficiencies (except carbohydrates), and nutritional imbalances were observed. This may be because GFD requires patients to avoid foods containing gluten. As a result, the data obtained for nutrient intake showed that the intake of CD females was very poor when compared to DRI. This finding is consistent with the Segura and Rosell [23] who reported that commercial GFD products often have the least nutritional value compared to gluten-rich ones.

Moreover, we observed that females with CD consume less fiber and micronutrients, such as iron, calcium, and vitamin D (VD), than what is recommended. Macronutrient intake for all participants was significantly low, especially fat, protein, and fiber intake, as compared to the DRI. Low fat and protein intake are not typical of Saudi dishes, indicating that this group is completely dependent on GFD. A similar result was reported by Larretxi et al. [24], who stated that GFD products contribute to an unbalanced diet. Additionally, Vici et al. [25] observed lower fiber intake in patients with CD than in healthy subjects. According to Younes et al. [26], the nutrient intake of CD patients, particularly VD, folate, and iron, should be evaluated, as this will cause bone disease as well as anemia. This restriction may lead CD patients to make choices such as eating foods high in calories, fat, and protein [3], which are expected to be linked to a higher risk of several diseases and overweight. 

There is an agreement that GFD restores intestinal villi and absorption function in celiac patients. As a result, the blood parameter values of the CD females who adhere to a long-term GFD should be comparable to those in the general population. With a few exceptions, the values obtained for blood tests in the current study were found to be within the reference ranges. The majority of the adult females with CD had a low PDW. It has been shown that PDW is a common test in blood routine examinations that reflects variation in platelet size distribution [27]. When platelets are activated in an inflammatory environment, they always undergo morphological changes. As a result, PDW can be used as a marker of activated platelets being released in some inflammatory diseases. PDW levels have been shown in some studies to change under specific conditions compared to healthy individuals [28]. All participants had a high level of PCT. Choi and McCarthy [29] reported that the PCT was applied as a reliable marker of severe bacterial infection. Since the majority of values fall within the reference ranges, we observed that there is no sign of abnormality in females with CD on a long-term GFD, except for the elevated level of PCT, VD deficiency, and anemia. In agreement with the present study, [30] describes common changes in patients with celiac disease, such as VD deficiency.

The results of anthropometric proxies showed that the majority of the CD adult females following GFD had a normal BMI, low WHR, decreased BF, decreased VF, and were slim. This finding can be explained by a significantly low intake of micro- and macronutrients compared to DRI and is consistent with the results of Bambrilla et al. [31], who showed a decreased weight gain and obesity rate in patients who depend solely on GFD. Despite relying solely on GFD, some patients were observed to gain weight and develop obesity. Tucker et al. [32] reported that BMI increases on GFD, especially that closely adherent and a significant proportion of CD patients were diagnosed with a high level of BMI. Further, they stated that females have a wider range of BMI and are more likely to be obese than males. Valletta et al. [33] showed an increased rate of weight gain in people with CD after one year of following GFD. However, one study found that GFD positively affected body composition, with BMI normalization in underweight and overweight subjects [34]. 

The significant positive correlation between the adult females’ independent variables and the dependent ones indicated that the independent factors favored the nutritional status of the females, such as age, type of residence, occupation, and marital status. However, some independent variables, such as the education level, adversely affected the nutritional proxies of the females. The significant adverse association between the females’ education level and the nutritional proxies could be because a less educated female is not aware of proper dietary habits. The study showed that depression and anxiety, the avoidance of social activities, and the cost of a GFD or inability to work adversely and significantly (*p* ≤ 0.05) influenced nutritional status. Addolorato et al. [35] reported that, along with the numerous gastrointestinal, nutritional, and metabolic consequences of CD, there has been a considerable concern about the increased rates of psychological symptoms and mental disorders in CD patients. CD patients have higher rates of depression than the general population controls, according to Ludvigsson et al. [36], which may affect their nutritional health. Addolorato et al. [37] found more anxiety in CD patients than in the controls, but Roos et al. [38] found no anxiety in CD patients. Decreased health-related quality of life in celiac disease is associated with comorbid physical and mental illness [39]. Joelson et al. [40] suggested that depression may alter the relationship between GFD adherence and the presence of CD symptoms. According to Arigo et al. [41], a significant subset of CD women who adhere to GFD have clinically relevant symptoms of depression and disordered eating; these symptoms are associated with increased psychosocial distress in other domains. Furthermore, Zysk et al. [42] reported that a CD patient’s economic status may be one of the most important factors influencing their social and emotional fears and worries, leading to a low level of social and emotional fears and worries. The higher relationship between BMI and other nutritional indices suggested that all or some of them could be used as indicators to assess the nutritional status of the participants.

The current research was based on dietary intake and body composition. However, there are some limitations, including that part of the study was cross-sectional, and accordingly, the results should be interpreted with caution, and it was difficult to include physical activity because the majority of respondents did not exercise. Finally, the sample size was low due to the small number of available cases because this study focuses on a specific region in Saudi Arabia, and it was difficult to cover the whole of Saudi Arabia with the study due to the size of the country.

## 5. Conclusions

Following a strict GFD for a long time had a large number of restrictions that could affect the quality of life and nutritional status of the females under investigation. When comparing the nutrient intake of celiac females to the dietary recommended intake (DRI), we observed a deficit in all nutrient intakes as a result of following GFD. Moreover, and as a result of a low intake of nutrients, the majority of adult females had a normal BMI, low WHR, decreased BF, decreased VF, and were slim. With a few exceptions, blood parameter values in females with CD who followed a GFD for a long time (>1 year) were comparable to those in healthy people. The educational level as a demographic factor and some psychosocial factors had the greatest impact on the participants’ nutritional status. These findings suggest that psychosocial care, in addition to existing dietary recommendations for people with celiac disease, have the potential to improve patient well-being.

## Figures and Tables

**Table 1:** Blood parameter and test results classification.

No.	Blood Parameter	Classification
1	White blood cells (WBC)	low < 4, normal 4–11, high > 11
2	Red blood cell (RBC)	low < 3.80, normal 3.80–6.50, high > 6.50
3	Hemoglobin (HGP)	low < 11.50, normal 11.50–17, high > 17
4	Hematocrit (HCT)	low < 36, normal 36–54, high > 54
5	Mean corpuscular volume (MCV)	low < 80, normal 80–96, high > 96
6	Mean corpuscular hemoglobin (MCH)	low < 26, normal 26–34, high > 34
7	Mean corpuscular hemoglobin concentration (MCHC)	low < 31, normal 31–37, high > 37
8	Platelet count (PLT)	low < 150, normal 150–400, high > 400
9	Red cell distribution width in fL (RDW-SD)	low < 37, normal 37–54, high > 54
10	Red cell distribution width in percentage (RDW-CV)	Low < 11, normal 11–16, high > 16
11	Platelet distribution width (PDW)	low < 12.2, normal 12.2–16.1, high > 16.1
12	Mean platelet volume (MPV)	low < 7.5, normal 7.5–11.4, high > 11.4
13	Platelet–large cell ratio (P-LCR)	low < 15, normal 15–30, high > 30
14	Procalcitonin test (PCT)	low < 0.042, normal 0.042–0.102, high > 0.102
15	Vitamin B12	low < 211, normal 211–963, high > 963
16	Vitamin D-T	low < 20, normal 20–32, high > 32

**Table 2:** Average daily intake of nutrients (24 h recorded—3 days) with the dietary requirement intake (DRI) for females with CD (*n* = 51) using the T-test.

Items Intake	Mean	DRI	Difference	T-Test	*p*-Value
Calories (kcal)	1769.00	2000.00	−231.00	−1.917 *	0.016
Protein (g)	40.72	46.00	−5.28	−2.29 *	0.026
Carbohydrates (g)	165.03	130.00	35.03	4.04 **	0.001
Dietary fiber (g)	10.35	28.00	−17.65	−15.69 **	0.000
Total fat (g)	47.41	65.00	−17.59	−5.24 **	0.003
Saturated fat (g)	15.84	20.00	−4.16	−4.05 **	0.007
Unsaturated fat (g)	32.36	45.00	−12.64	−5.11 **	0.005
Cholesterol (mg)	156.80	300.00	−143.20	−10.34 **	0.000
Vit A µg (RE)	191.80	700.00	−508.20	−6.07 **	0.010
Vit B1 (mg)	0.62	1.10	−0.48	−2.64 *	0.011
Vit B2 (mg)	0.59	1.10	−0.51	−6.43 **	0.000
Niacin (mg)	7.01	14.00	−6.99	−11.15 **	0.008
Vit B6 (mg)	0.54	1.30	−0.76	−8.55 **	0.000
Vit B12 (mg)	0.96	2.40	−1.44	−3.22 **	0.002
Vit E (mg)	1.78	15.00	−13.22	−84.36 **	0.000
Folate (mg)	101.10	400.00	−298.90	−37.05 **	0.005
Calcium (mg)	438.49	1000.00	−561.51	−14.31 **	0.001
Copper (mg)	0.50	900.00	−899.50	−5134.28 **	0.000
Iron (mg)	5.91	18.00	−12.09	−25.22 **	0.003
Phosphorus (mg)	289.84	700.00	−410.16	−18.56 **	0.001
Selenium (mg)	28.13	55.00	−26.87	−10.47 **	0.000
Zinc (mg)	2.28	11	−8.72	−41.97 **	0.004

** *p* ≤ 0.01; * *p* ≤ 0.05; Difference = mean–DRI.

**Table 3:** Basic hematological parameters of females with CD (*n* = 51).

Variables	LOW		NORMAL		HIGH			
	Frequency	Percent	Frequency	Percent	Frequency	Percent	Chi-Square	*p*-Value
WBC (10^3^/μL)	4	7.8	46	90.2	1	2.0	74.471 **	0.002
RBC (10^6^/μL)	2	3.9	49	96.1	__	__	43.342 **	0.005
HGP (g/dL)	23	45.1	28	54.9	__	__	0.490	0.484
HCT (%)	19	37.3	32	62.7	__	__	3.314	0.069
MCV (fl)	20	39.2	31	60.8	__	__	2.373	0.123
MCH (pg)	21	41.2	30	58.8	__	__	1.588	0.208
MCHC (g/dL)	19	37.3	32	62.7	__	__	3.314 *	0.043
PLT (10^3^/μL)	__	__	41	80.4	10	19.6	18.843 **	0.004
RDW-SD (fL)	1	2.0	50	98.0	__	__	47.007 **	0.001
RDW-CV (%)	1	2.0	36	70.6	14	27.5	36.824 **	0.003
PDW (fL)	49	96.1	2	3.9	__	__	43.314 **	0.001
MPV (fL)	__	__	50	98.0	1	2.0	47.078 **	0.006
P-LCR (%)	2	3.9	47	92.2	2	3.9	79.412 **	0.001
PCT (%)	__	__	__	__	51	100.0	__	__
B12 (pg/mL)	0	0.0	51	100.0	__	__	__	__
VITD-T (ng/mL)	28	54.9	19	37.3	4	7.8	17.294 **	0.0001

** *p* ≤ 0.01; * *p* ≤ 0.05.

**Table 4:** Body mass index (BMI), waist-to-hip ratio (WHR), body fat (BF), visceral fat (VF), and waist-to-height ratio (WHtR) of females with CD (*n* = 51).

Anthropometric	Frequency	Percentage
*Body mass index (BMI)*		
Underweight	5.0	9.8
Normal	26.0	51.0
Overweight	14.0	27.5
Obesity I	2.0	3.9
Obesity II	4.0	7.8
Obesity III	0.0	0.0
Total	51	100.0
*Waist-to-hip ratio (WHR)*		
Low	26	51.0
Normal	3	5.9
High	22	43.1
Total	51	100.0
*Body fat (BF)*		
Decreased	22	43.1
Normal	19	37.3
High	3	5.9
Very high	7	13.7
Total	51	100.0
*Visceral fat (VF)*		
Decreased	31	60.8
Normal	13	25.5
High	7	13.7
Very high	–	–
Total	51	100.0
*Waist-to-height ratio (WHtR)*		
Extremely slim	3	5.9
Slim	17	33.3
Normal	13	25.5
Overweight	3	5.9
Too overweight	15	29.4
Total	51	100.0

BMI: chi-square = 35.373 (*p*-value = 0.001). Waist–Hip Ratio: chi-square: 17.765 (*p*-value = 0.0013). BF: chi-square = 8.941 (*p*-value = 0.011), VF: chi-square = 18.353 (*p*-value = 0.003). WHtR: chi-square = 17.725 (*p*-value = 0.001).

**Table 5:** Spearman correlation coefficients and simple linear regression analysis between demographic characteristics and waist-to-hip ratio, waist-to-height ratio, body mass index (BMI), body fat (BF), and visceral fat (VF) of females with CD (*n* = 51).

Independent Variable/ Dependent Variable	WHR		WHtR		BMI		BF		VF	
	rho	(β, SE)	rho	(β, SE)	Rho	(β, SE)	rho	(β, SE)	r	(β, SE)
Age	0.597 **	(2.870 **, 0.271)	0.449 **	(3.836 **, 0.201)	0.459 **	(0.907 **, 0.177)	0.498 **	(0.620 **, 0.239)	0.351 **	(1.316 **, 0.205)
Type of residence	0.022 **	(0.282 **, 00.169)	0.427 **	(1.028 **, 0.182)	0.311 *	(0.335 *, 0.097)	0.139	(0.088, 0.019)	0.002	(0.003, 0.007)
Occupation	0.669 **	(0.670 **, 0.724)	0.779 **	(7.151 **, 0.668)	0.909 **	(2.081 **, 0.825)	0.898 **	(1.212 **, 0.807)	0.768 **	(2.371 **, 0.589)
Marital status	0.347 *	(1.679 *, 0.103)	0.261	(0.014, 0.069)	0.332 *	(0.026 *, 0.072)	0.376 *	(0.019 *, 0.112)	0.421 **	(0.056 **, 0.179)
Education level	−0.106 *	(−1.098 *, 0.128)	−0.356 *	(0.284 *, 0.205)	−0.237 *	(−0.042 *, 0.113)	−0.040 *	(−0.015 *, 0.120)	−0.017 *	(−0.022 *, 0.107)

* *p* ≤ 0.05, ** *p* ≤ 0.01, (rho) Correlation Coefficient/Regression coefficients (β), standard error (SE).

**Table 6:** Pearson correlation coefficients between some psychosocial queries and body mass index (BMI), body fat (BF), and visceral fat (VF) of females with CD (*n* = 51).

Independent Variable/ Dependent Variable	BMI		BF		VF	
	*p*-Value	(β, SE)	*p*-Value	(β, SE)	*p*-Value	(β, SE)
Have you ever been concerned about the emergence of a health issue related to celiac disease?	0.000	0.987 **, 1.027	0.029	1.747 *, 1.641	0.050	0.015 *, 0.798
Have your daily activities been restricted due to celiac disease?	0.377	0.116, 1.123	0.884	0.264, 1.794	0.522	0.564, 0.873
Have you felt that others kept you away from attending social activities?	0.918	1.705, 1.146	0.231	2.234, 1.832	0.779	0.252, 0.892
Did you feel depressed and anxious?	0.046	−0.149 *, 0.907	0.061	−0.642 *, 1.450	0.240	−0.842, 0.706
Have you been bothered by your weight?	0.870	−0.802, 0.806	0.381	−1.143, 1.288	0.471	−0.456, 0.627
Did you avoid social activities?	0.026	−1.118 *, 1.030	0.013	−2.749 *, 1.646	0.678	−0.335, 0.801
Have you been annoyed or frustrated about the cost of alternative gluten-free foods?	0.285	1.752, 1.290	0.027	3.825 *, 2.063	0.019	1.408 *, 1.004
Have you faced financial difficulties as a result of your coeliac disease (e.g., the cost of a gluten-free diet or inability to work)?	0.038	−0.037 *, 1.303	0.081	−0.507*, 2.083	0.315	−1.033, 1.013
Have you had trouble finding appropriate food?	0.978	−1.112, 1.028	0.481	−1.169, 1.643	0.912	−0.089, 0.799
Have you ever had to skip meals or snacks due to a lack of suitable food?	0.286	0.272, 0.926	0.301	1.553, 1.480	0.489	0.503, 0.720
Have you experienced fatigue or a lack of energy that you believe is caused by celiac disease?	0.771	1.123, 1.119	0.290	1.919, 1.788	0.628	0.618, 0.870
Have you been worried that you might accidentally eat or drink products containing gluten?	0.022	0.559 *, 1.181	0.002	0.832 **, 1.888	0.042	0.449 *, 0.919

* *p* ≤ 0.05, ** *p* ≤ 0.01, (r) Correlation Coefficient/Regression coefficients (β), standard error (SE).

## Data Availability

The datasets used and analyzed during the current study are available from the corresponding author upon reasonable request.
